# Coupling and confinement of current in thermoacoustic phased arrays

**DOI:** 10.1126/sciadv.abb2752

**Published:** 2020-07-01

**Authors:** David M. Tatnell, Mark S. Heath, Steven P. Hepplestone, Alastair P. Hibbins, Samuel M. Hornett, Simon A. R. Horsley, David W. Horsell

**Affiliations:** Department of Physics and Astronomy, University of Exeter, Stocker Road, Exeter EX4 4QL, UK.

## Abstract

When a medium is rapidly heated and cooled, heat transfers to its surroundings as sound. A controllable source of this sound is realized through joule heating of thin, conductive films by an alternating current. Here, we show that arrays of these sources generate sound unique to this mechanism. From the sound alone, we spatially resolve current flow by varying the film geometry and electrical phase. Confinement concentrates heat to such a degree that the film properties become largely irrelevant. Electrical coupling between sources creates its own distinctive sound that depends on the current flow direction, making it unusually sensitive to the interactions of multiple currents sharing the same space. By controlling the flow, a full phased array can be created from just a single film.

## Teaser

The interaction of currents in arrays of acoustic elements based on joule heating creates a unique, controllable sound source.

## INTRODUCTION

Arrays of speakers can be used to shape the sound they create in the air around them. An ever-increasing number of applications are taking advantage of this; these include medical diagnostics (*1*), acoustic levitation (*2*), wireless power transfer (*3*), photoacoustic imaging (*4*), acoustic holography (*5*), and fault detection in materials (*6*). The arrays used in all cases rely on modulation of the air volume to produce or detect the sound. Thermoacoustics is the production of sound through the modulation of air temperature. Natural examples include thunder (*7*), the roar of a fire (*8*), stellar oscillations (*9*), and the popping sounds made by bombardier beetles (*10*). Joule heating of thin, electrically conductive films or wires by an alternating current provides a laboratory-based paradigm of this effect (*11*, *12*). The source is nonresonant, broadband, and, for single-drive frequencies, has high fidelity (*13*, *14*). Hence, arrays of these sources (*15*–*17*) could offer considerable advantages for many array-based technologies, if phase control of individual elements could be realized. Of particular note is that the elements only act as sources, not receivers: Coupled with the fact that the production mechanism involves no mechanical movement of the source, this transcends issues associated with speaker-microphone cross-talk (*18*). Elements of a thermoacoustic-phased array might be expected to be uncoupled, as the transduction is a one-way thermodynamic process (*19*). We find that this is not the case: The elements are intrinsically electrically coupled. Such mutual coupling is known to be a problem for electromagnetic arrays (*20*); however, for thermoacoustic arrays, it is far from detrimental. The coupling creates an unusual “phantom” source of sound, with a distinctive and highly adaptable character. This can be exploited to radically simplify phased array design and facilitate techniques such as voltage-controlled vortex beam steering.

## RESULTS

**Fig. 1:**
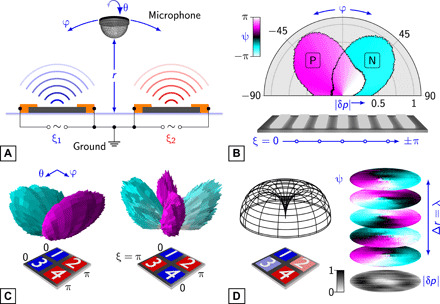
Sound generation from a thermoacoustic phased array. (**A**) Schematic of a two-element array. (**B**) Sound measured from a graphene-based seven-element array with incremental phase difference: Δξ = ±π/6 resulting in P and N, respectively. The normalized sound pressure, |δ*p*|, (*f* = 16 kHz) is shown as a function of angle, φ, across the array at a fixed distance, *r*, from its center (*r* = 0.16 m). (**C**) Dipolar (left) and quadrupolar (right) sound measured from a graphene-based 2 × 2–element array. The sound field is shown above the schematic. In (B) and (C), radial length depicts the magnitude of δ*p*, and the fill color depicts its phase, ψ. (**D**) Source phases were incremented in a clockwise manner (1, 2, 4, 3) = (0, π/2, π, 3π/2). The resulting sound magnitude (simulated) is shown above the schematic. Orthographic projections (right) of the resulting experimental |δ*p*| (bottom) and ψ(*r*) (stack) are shown in tilted perspective.

Thermoacoustic phased arrays allow precise electrical control of the acoustic field. Sound was measured over a hemispherical surface in the air above a wide variety of array types (Fig. 1A, fig. S1, Materials and Methods, and text S1). Control of the array was demonstrated through steering a beam of sound using a seven-element linear array. The phase of the joule power dissipated in each element was offset stepwise across the array (Fig. 1B). The resulting sound was steered in a direction away from the array normal, the phase offset being equivalent to physically tilting the array surface. A two-dimensional planar array adds a further degree of control to the sound output. With two different phases used in a 2 × 2–element array, dipolar and quadrupolar sound fields were created (Fig. 1C). With three or more phases, the sound field included finite vorticity (Fig. 1D) (*21*). This was seen as a distinctive twist in the phase profile (*22*) that rotated about a singularity at its center as a function of distance, *r*, from the array, its pitch equaling the acoustic wavelength, λ.

**Fig. 2:**
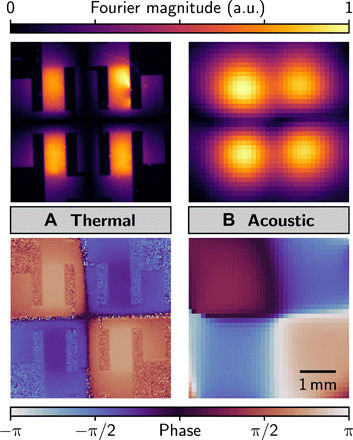
Transforming sound into joule heat. Radiated heat and joule heat were measured from a graphene-based 2 × 2–element array (fig. S1A) driven as a quadrupolar source. a.u., arbitrary units. (**A**) Thermal reconstruction of the array from the Fourier component at the second harmonic of the drive frequency (1 Hz). (**B**) Acoustic reconstruction from far-field sound measurements at 300 kHz. In both reconstructions, the magnitude (top) and phase (bottom) are shown using the same color and spatial scales.

A detailed picture of the array is formed by considering its electrical, thermal, and acoustic properties together. The far-field sound (*r* ≫ λ) is the Fourier transform of its source. We used this to reconstruct the magnitude and phase of the joule heating across the array surface (Fig. 2, fig. S2, and text S2). A thermal camera was used to record the radiated heat from a 2 × 2–element array driven as a quadrupolar source at a low frequency. The thermal reconstruction of the magnitude and phase of the radiated heat was derived from a temporal Fourier transform of the recorded image sequence (Fig. 2A). The inverse spatial Fourier transform of the far-field sound measured at 300 kHz (cf. Fig. 1C) resulted in an acoustic reconstruction of the joule heat (Fig. 2B). Aside from a difference in diffraction-limited resolution, the acoustic and thermal reconstructions agreed in their salient features; however, the former did not suffer from the effect of low emissivity, typical of metal films (which causes the gold electrodes to have low signal-to-noise ratio in Fig. 2A).

**Fig. 3:**
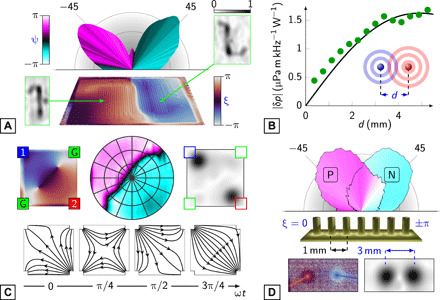
Types of thermoacoustic array. (**A**) Measured beam profile of the dipolar sound from two elements of a graphene-based 16-element array (top) and reconstructed thermal phase of the array surface (bottom) shown in tilted perspective. Insets show acoustic reconstructions of individual elements (fig. S1A). (**B**) Integrated |δ*p*| measured from a two-element dipole as a function of element separation (circles). The result from an analytical model of two point sources is also shown (line). (**C**) A 6-mm square indium tin oxide film run as a dipole: sources (1, 2) and grounds (G). Top: The measured thermal phase (left), orthographic projection of the far-field sound phase (middle), and acoustic magnitude reconstruction (right). Bottom: Stream plots of the current (from numerical modeling) as it moves between the electrodes. (**D**) Measured beam steering by a seven-element gold junction array, to compare directly with Fig. 1B. Bottom: Thermal phase (left) and acoustic magnitude (right) reconstructions of a two-element junction dipole.

The fact that sound results from the power dissipated in a resistive medium affords two distinct ways to realize a phased array (Fig. 3). The first, conventional way would be to form it from separate resistive elements (Figs. 1, B to D, and 3A). The total sound output from pairs of such elements was found to decrease as their separation was reduced, in agreement with a simple model of point-like sources (Fig. 3B and text S3). The second, unorthodox way would be to use a single resistive film and apply multiple voltages to electrodes connected to it. A square film connecting four electrodes at its corners, with two voltage sources set π/2 out of phase and the others grounded, produced a sound field with a strong dipolar character (Fig. 3C). The reason this worked is to be found in the acoustic reconstruction: The joule power was concentrated in small regions around the electrodes with little sound being produced from the bulk of the film. One might suspect this to be the result of poor mechanical contact, leading to a high junction resistance. We found that this was not the case.

The high joule power dissipation around electrodes is caused by bottlenecks in the current flow. This power, proportional to the density of the current, is highly focused around such constrictions (Fig. 3C and text S4) (*23*). The sharing of power between the bottlenecks is dictated by the flow of the current over a cycle of the source voltage: The current driven into the film periodically switches between draining into the grounds and into the other source. In this particular geometry, a much higher proportion of the “current crowding” occurs around the two sources than the grounds, resulting in the observed dipolar sound field.

Current crowding is such a dominant effect that it can be used to create a phased array. We demonstrated this by constructing an array of 25-μm-diameter gold wires, spaced 1 mm apart, and bound to the surface of a 50-nm-thick gold film (Fig. 3D). This diameter is comparable to the smallest piezoelectric phased array elements (*24*). The current has to diverge from the base of each wire, and its density decreases with distance into the film (text S4). Therefore, effectively, all the joule power was dissipated within a small distance (≪1 mm) from the wire-film junction. This was confirmed by an acoustic reconstruction of a dipole formed by two of the wires (Fig. 3D). Extending this idea further, a multijunction array of wires was used to steer an acoustic beam in the same way as a separate-element array (cf. Fig. 1B).

**Fig. 4:**
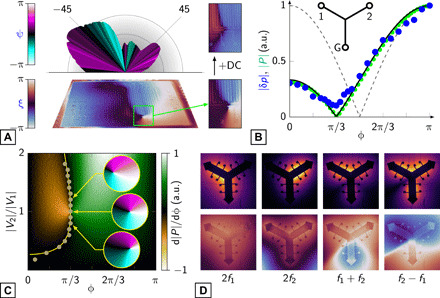
Phantom sound sources. (**A**) Measured beam profile and thermal phase reconstruction of a dipole formed by two elements of a 16-element array that share a common ground trace. Left: A zoomed region (right) showing the vortex formed at the element-trace junction and its suppression by the addition of a DC current. (**B**) Integrated sound pressure (blue) and joule power (green) as a function of the phase difference between two sources in a zinc-based three-branch array (inset): experiment (circles) and theory (line) for the coupled case and theory (dashed line) for the uncoupled. (**C**) Total absolute joule power from a three-branch array measured as a function of voltage ratio and phase difference; experimental (circles) and theoretical (line) path of minimum power. Insets: simulated orthographic projections of the sound phase at ϕ = π/3 and different voltage ratios. (**D**) Measured thermal magnitude (top) and phase (bottom) reconstructions of the three-branch array in the case *f*_2_ = 4*f*_1_.

Currents that are linked in phase must inherently share a physical connection. This leads to unavoidable coupling in a thermoacoustic array. To investigate this, we measured the acoustic output of two elements that shared a “trace” ground electrode (Fig. 4A, fig. S3, and text S5). A trace connecting many elements to a single ground point is a common, effective method to reduce the total number of electrodes needed to address multielement arrays. Each element was a short, wide strip of graphene (0.2 mm by 6 mm) sandwiched between two strips of gold. Hence, each element only had a resistance of a few ohms. It is tempting to consider the trace as an electrical and thermal reservoir, large enough to absorb all charge and heat drained into it. However, the trace in a real circuit is not a perfect conductor [an important issue in high-frequency electronics; (*25*)] and, in our case, has a resistance less than an order of magnitude smaller than that of the elements. Hence, the trace contributes to the sound output (seen as a small offset in the integral sound; Fig. 3B). When the sources are run as an acoustic monopole, a common trace carries both source currents, and its contribution can be substantial, even dominant (fig. S3, B and C). Run as a dipole, its contribution is smaller but more complex.

The nonlinear nature of the coupling between array elements leads to additional sources of sound. In the case of a two-element dipole with a common trace, the source created by the coupling added a third phase component to the joule power. This caused a thermal vortex around the junction between the trace and the element closest to the ground point (Fig. 4A), which was found to be negated only by application of a DC voltage to the sources (text S6). Such “phantom” sources are not confined to any particular region of an array. To illustrate this, we created a single film shaped into three branches of equal resistance, *R*, that radiated out from a common center (Fig. 4B, fig. S4, and text S7). One branch was grounded, and separate oscillatory voltages, *V*_1_ and *V*_2_, were applied to the other two with a phase difference, ϕ, between them. Voltage probes located along each branch allowed the currents, *I*_1_ and *I*_2_, and, consequently, the joule power to be measured. For an uncoupled system, the total sound resulting from $I_{1}^{2}R$ and $I_{2}^{2}R$ would be equal at ϕ = 0 and π and minimal at π/2 (the acoustic dipole). Coupling distorts this: Currents from the two sources at ϕ = 0 both flow into the ground, but at ϕ = π, flow from one source into the other. The presence of the phantom, which is proportional to *I*_1_*I*_2_*R*, is felt through its effect of shifting the phase of the total joule power and sound pressure minima from π/2 to π/3 (Fig. 4, B and C, and texts S8 and S9).

The phantom source has its own distinctive acoustic character. In the three-branch array, its effect was to create net vorticity in the sound around ϕ = π/3 (Fig. 4C). This vortex could be steered simply through the balance of voltages applied to the two sources. To understand the nature of the phantom, it was investigated independently by spectrally isolating it from the two original sources. This was achieved in our three-branch array by setting the voltage sources at different frequencies, *f*_1_ and *f*_2_. The phantom then uniquely occurred at the sum and difference heterodynes of these frequencies (*12*), ∣*f*_1_ ± *f*_2_∣ (Fig. 4D, fig. S4D, and text S10). The phantom had a sound intensity equal to that of its two sources; however, in contrast to these monopolar sources, the phantom had a dipolar character. Also, the dipole that formed at the sum heterodyne is the inverse of that at the difference heterodyne. This is a result of energy conservation. The coupling requires energy to be transferred between the two source branches and the ground branch. These energy changes must be equal and opposite; hence, the joule heating in the source and ground branches is out of phase. This creates the dipolar sound fields observed at the frequencies of the phantom source.

## DISCUSSION

As the phantom sound source depends on the ratio of source voltages, element resistances, phase difference, and frequency, it ultimately forms the most flexible source of sound in a thermoacoustic array (Fig. 4, C and D). Combined with the effect of confinement (Fig. 3, C and D), which affords geometric control of the sound, such arrays offer opportunities that are not available in established phased array technologies. The facile, economical, and scalable fabrication methods, with potential to miniaturize down to the nanoscale and to integrate mechanical flexibility and optical transparency into the design, provide a practical route to next-generation audiovisual devices where a transparent electrode could double as both audio source and optical window [although, currently, the efficiency of transduction needs to be improved by several orders of magnitude for this to be practical (*12*)]. In particular, where flexing would cause distortion of the sound field (*26*–*28*), fine electrical control would permit simple compensation.

The real potential of thermoacoustic phased arrays lies beyond audio reproduction. They allow us to answer some fundamental questions about sound generation. The output from a dipolar sound (or electromagnetic) source decreases to zero when the sources are brought together (cf. Fig. 3B). However, to have a phase difference inherently requires a common reference (a ground, for example). As a result, the phantom source produced by the nonlinear coupling through this reference remains finite even when the separation of the sources is zero. In the cases we examined, this phantom source is monopolar (Fig. 4D), but questions about the nature and proximity of the reference remain to be answered.

From a practical perspective, banishing the phantom through measurement of the sound could become a key method in designing more thermally efficient electrical circuits (Fig. 4, B and C). This direct link it has to the coupling, and the fact that its sound field is distinct from that of its sources, presents unforeseen possibilities in covert communication and medical imaging technologies. We have shown that the sound from thermoacoustic arrays can be further controlled through current crowding to such an extent that phase control is possible in a single conductive film (Fig. 3, C and D). This offers a previously unexplored route to investigate phase-driven control of crowding effects in other physical and biological systems. The dominance of confinement and coupling effects at small physical scales suggests that submicron-sized phased arrays can only be realized through phantom sound generation from heat at electrical constrictions.

## MATERIALS AND METHODS

Linear and planar arrays of thermoacoustic thin-film sources were fabricated (text S1) and measured in the frequency range of 1 Hz ≤ *f* ≤ 300 kHz. These arrays were composed of up to 16 elements, varying in sizes between 0.2 and 20 mm (fig. S1). The thin film forming the elements was either graphene, indium tin oxide, zinc, or gold; all connecting electrodes were gold. Elements were powered individually from amplified, phase-locked sources (Signal Recovery). By this, the amplitude and relative phase of the current supplied to each element could be controlled. The sound was detected over a frequency range from 2 to 300 kHz by a condenser microphone (Earthworks) separated from the device at distances ranging from 0.05 to 1 m. The microphone signal was measured by a lock-in amplifier locked to the source frequency. Devices were mounted on an adapted gimbal stage (Newmark Systems) and rotated in two orthogonal directions with respect to the fixed microphone. Thermal measurements were made with a thermal camera (FLIR Systems Inc.) in the frequency range from 1 to 100 fps. All instruments were controlled, and data were taken using CryoMeas software for Acorn RiscPC (C. J. B. Ford, University of Cambridge); analytical modeling and all analysis were performed using Python; COMSOL Multiphysics (COMSOL Inc.) was used for finite-element modeling.

## Supplementary Material

abb2752_SM.pdf

## SUPPLEMENTARY MATERIALS

Supplementary material for this article is available at http://advances.sciencemag.org/cgi/content/full/6/27/eabb2752/DC1
