# Supplementary material for: Coupling and confinement of current in thermoacoustic phased arrays
Source: Sci Adv. 2020 Jul 1;6(27):eabb2752. doi: 10.1126/sciadv.abb2752 (PMC7329335; doi:10.1126/sciadv.abb2752)
Supplement: abb2752_SM.pdf [file abb2752_SM.pdf]

Science Advances
AAAS

advances.sciencemag.org/cgi/content/full/6/27/eabb2752/DC1

Supplementary Materials for

Coupling and confinement of current in thermoacoustic phased arrays

David M. Tatnell, Mark S. Heath, Steven P. Hepplestone, Alastair P. Hibbins, Samuel M. Hornett, Simon A. R. Horsley, David W. Horsell*

*Corresponding author. Email: d.w.horsell@exeter.ac.uk

Published 1 July 2020, *Sci. Adv.* **6**, eabb2752 (2020)
DOI: 10.1126/sciadv.abb2752

This PDF file includes:

Texts S1 to S10
Figs. S1 to S4

S1. DEVICE FABRICATION

The film forming the active element of the thermoacoustic devices was either CVD-grown monolayer graphene (commercially sourced from Graphene Laboratories Inc., Graphene Square Inc. and Graphenea Inc.), indium tin oxide (ITO; Testbourne Ltd), zinc or gold. The films were deposited on $p^{+}Si(300\,nm)/SiO_2$ (IDB Technologies Ltd), quartz (UQG Ltd) or glass substrates. All devices were patterned using electron-beam lithography (Nanobeam NB4). Around 18 devices were used for the measurements in this work. Various device architectures are shown in fig. S1A. Within the parameter space of the measurements, the principal features of the results were found to be independent of film and substrate material type.

Graphene-based devices were patterned and subsequently dry etched in a reactive ion etcher (JLS RIE80). The plasma power was 30 W and the process gases were oxygen (10 sccm) and argon (10 sccm) at a partial pressure of 15 mTorr. Electrodes were deposited via thermal evaporation (Edwards 306) in two steps: Au only (50 nm) on the graphene followed by Cr(5 nm)/Au(100 nm) overlapping the Au and extending onto the substrate surface.

For the ITO-based devices, the ITO film was produced via sputter deposition (Moorfield NanoPVD) at 30 W RF power and a process pressure of 5×10^{-1} mbar using 99.999% purity argon gas. Electrodes were deposited in a single step of Cr(5 nm)/Au(120 nm). For the zinc-based devices, a zinc film was deposited via thermal evaporation at a rate of 1 Å/s and base pressure of 2×10^{-6} mbar to achieve a 30 nm thick film. Electrodes were deposited as for the ITO devices. For the gold junction devices, a 50 nm gold film was deposited via thermal evaporation with 5 nm Cr used as a sticking layer.

All devices were mounted in 44-pin ceramic leadless chip carriers (Spectrum Semiconductor Materials Inc.) using conductive silver paint or superglue. The electrodes on the devices were wedge- or ball-bonded to the pins of the carrier using 25 μm gold wire (K&S 4700 wedge bonder, Westbond 7700E ball bonder).

S2. THERMOACOUSTIC RESPONSE

In modelling the thermoacoustic response, we need to consider the heat input into the system via Joule heating, δQ_{in}, and that lost to the air in the form of sound, δQ_{air}. A useful material parameter to consider is the effusivity,

$$e = \sqrt{\kappa\rho c_p}\,,$$

where κ is the thermal conductivity, ρ is the density and c_p is the heat capacity. It can be shown that (*12*),

$$\delta Q_{\mathrm{air}} = e_{\mathrm{r}}\delta Q_{\mathrm{in}}\,.$$

where the relative effusivity of the air to the whole system,

$$e_{\mathrm{r}} = \frac{e_{\mathrm{air}}}{e_{\mathrm{air}} + e_{\mathrm{substrate}} + e^{*}_{\mathrm{film}}} \approx \frac{e_{\mathrm{air}}}{e_{\mathrm{substrate}}}\,.$$

Here e^{*}_{film} is an effusivity-like term accounting for the absorption of energy by the film. The approximation is valid for typical materials and frequencies (including those used in this work) as $e_{\mathrm{substrate}}$ exceeds $e_{\mathrm{air/film}}$ by several orders of magnitude.

The Joule power, $P = \delta Q_{\mathrm{in}} f$, where f is the second harmonic frequency of the source voltage/current. As no mechanical work is done by the film, the pressure variation in the air (*i.e.* the sound), $\delta p = \delta Q_{\mathrm{air}}/V$, where V is the volume of air heated per cycle. For a point-like source, this volume,

$$V = \frac{2\pi}{3}\left(\frac{v_{\mathrm{air}}}{f}\right)^{3}\,,$$

where v_{air} is the speed of sound. The pressure is maximal at $r_0 = v_{\mathrm{air}}/2f$ and decreases with the inverse of the distance, r, from the source. As such, the sound pressure,

$$\delta p \approx \frac{3e_{\mathrm{r}}}{4\pi v_{\mathrm{air}}^{2}}\frac{fP}{r}\,.$$

This equation links the power input into the film by an AC electrical supply to the sound generated by it, fig. S1B.

For phased arrays, we consider the far-field pressure variation in the air as a spherical wave centred on a particular source:

$$\delta p'(\boldsymbol{r},\boldsymbol{r}',t) \approx A\frac{\mathrm{e}^{i\left(\boldsymbol{k}\cdot(\boldsymbol{r}-\boldsymbol{r}')-\omega t\right)}}{|\boldsymbol{r}-\boldsymbol{r}'|}\,.$$

Here, $A = 3e_{\mathrm{r}}fP/4\pi v_{\mathrm{air}}^{2}$, $\boldsymbol{k}$ is the wavevector, and $\boldsymbol{r}'$ and $\boldsymbol{r}$ are the displacements of the source and detector from the origin, respectively. The source power

$$P = P_0\cos(2\pi ft + \xi)\,,$$

results from Joule heating by a current driven through the film. The phase ξ accounts for relative phase shifts between the different source elements.

The array surface can be considered as a distribution of point-like emitters at positions $\boldsymbol{r}' = (x', y')$, each with an associated source power $P(\xi)$. The total acoustic pressure at position $\boldsymbol{r} = (x, y, z)$ is determined from integrating over the array surface S. In the half-space above the emitters ($z > 0$),

$$\delta p(\boldsymbol{r}) = \int_S \delta p'(\boldsymbol{r}, \boldsymbol{r}')\, \mathrm{d}\boldsymbol{r}'\,.$$

In the far-field ($|\boldsymbol{r}| \gg |\boldsymbol{r}'|$), the pressure in spherical polar coordinates can be approximated as

$$\delta p(r, \varphi, \theta) \approx \frac{\mathrm{e}^{ikr}}{r} \iint_{-\infty}^{\infty} A \mathrm{e}^{i\left(k_x x' + k_y y'\right)}\, \mathrm{d}x' \mathrm{d}y'\,. \quad \text{(S1)}$$

Therefore, the far-field acoustic pressure is the 2D Fourier transform of the amplitude distribution $A(x', y')$. By measuring both the magnitude and phase of the far-field acoustic pressure over the hemisphere enclosing the array, we can reconstruct the amplitude distribution via an inverse Fourier transform, fig. S2B.

S3. INTEGRATED SOUND PRESSURE FROM A DIPOLE

Consider two point sources, with strengths $Q_{1,2}$, wavevectors $k_{1,2}$ positions $\boldsymbol{r}_{1,2}$ and phase difference ψ. The time-averaged sound pressure at a point $\boldsymbol{r}$ is

$$\begin{aligned}\delta p(\boldsymbol{r}) = &- i\frac{Z}{4\pi}\left(\frac{Q_1 k_1}{|\boldsymbol{r} - \boldsymbol{r}_1|}\mathrm{e}^{ik_1|\boldsymbol{r}-\boldsymbol{r}_1|}\right.\\ &+ \left.\frac{Q_2 k_2}{|\boldsymbol{r} - \boldsymbol{r}_2|}\mathrm{e}^{i(k_2|\boldsymbol{r}-\boldsymbol{r}_2| + \psi)}\right)\,,\end{aligned}$$

where Z is the acoustic impedance of the medium. The sound power, W, radiated into a solid angle, Ω, is defined as

$$W = \iint_\Omega \frac{|\delta p|^2}{Z}\, \mathrm{d}\Omega\,,$$

where $|\delta p|^2 = \delta p \delta p^*$. In spherical coordinates,

$$W = \int_0^{2\pi}\int_0^{\pi} \frac{|\delta p|^2}{Z} r^2 \sin\theta\, \mathrm{d}\theta\, \mathrm{d}\varphi\,.$$

If the two point sources on the z-axis are located at $\pm d/2$, then in the far-field ($|\boldsymbol{r}| \gg |\boldsymbol{r}_x|$),

$$\begin{aligned}|\boldsymbol{r} - \boldsymbol{r}_x| &\approx |\boldsymbol{r}| - \boldsymbol{r}_x \cdot \hat{\boldsymbol{r}}\\ &\approx r - z\cos\theta\,.\end{aligned}$$

The sound pressure becomes

$$\begin{aligned}\delta p(r, \theta, \varphi) = &- i\frac{Z}{4\pi r}\left(Q_1 k_1 \mathrm{e}^{ik_1 r}\mathrm{e}^{-ik_1 d\cos\theta/2}\right.\\ &+ \left. Q_2 k_2 \mathrm{e}^{ik_2 r}\mathrm{e}^{ik_2 d\cos\theta/2}\mathrm{e}^{i\psi}\right)\end{aligned}$$

and the sound power

$$\begin{aligned}W = \frac{Z}{16\pi^2}\int_0^{2\pi}\int_0^{\pi} &\left(Q_1^2 k_1^2 + Q_2^2 k_2^2\right)\sin\theta\\ &+ Q_1 Q_2 k_1 k_2 \sin\theta\left[\mathrm{e}^{i(k_2-k_1)r}\mathrm{e}^{i(k_1+k_2)d\cos\theta/2}\mathrm{e}^{i\psi}\right.\\ &+ \left.\mathrm{e}^{-i(k_2-k_1)r}\mathrm{e}^{-i(k_1+k_2)d\cos\theta/2}\mathrm{e}^{-i\psi}\right]\, \mathrm{d}\theta\, \mathrm{d}\varphi\,,\end{aligned}$$

Evaluating this integral, we finally obtain

$$\begin{aligned}W = &\frac{Z}{4\pi}\left\{\left(Q_1^2 k_1^2 + Q_2^2 k_2^2\right)\right.\\ &+ 2Q_1 Q_2 k_1 k_2 \cos\left((k_2 - k_1)\, r + \psi\right)\\ &\times \left.\mathrm{sinc}\left(\frac{(k_1 + k_2)d}{2}\right)\right\}.\end{aligned}$$

The square-root of W, which is proportional to $|\delta p|$, is used in the model (solid line) shown in Fig. 3B.

S4. CURRENT CROWDING

Consider a wire of radius r_1 and conductivity σ_1 running parallel to the z-axis. It joins a thin film in the xy-plane of thickness $t \ll r_1$ and conductivity σ_2. Assume that at some height above the film the wire is cut and its free end is held at potential V_1. Similarly, assume the film is cut into a large circle of radius r_2 centred on the wire, and the circle edge is held at potential V_2. The potential difference, $V_1 - V_2$, is such that current is driven down the wire and out to the edge of the film.

At the free end of the wire, the electric field points along $-\hat{z}$ and has a uniform value over the cross section of the wire. By Ohm's law, $\boldsymbol{j}_1 = \sigma_1 \boldsymbol{E}_1$, the current must also be uniform across the wire. If the wire carries total current I, then at the free end the current density will have the form:

$$\boldsymbol{j}_1 = -\frac{I}{\pi r_1^2}\hat{\boldsymbol{z}}\,.$$

If the wire was infinitely long, then this expression would hold all along the wire. However, in this case it will only be true up to the vicinity of the junction between it and the film, where the current has to change direction.

The electric field at the edge of the film ($r = r_2$) will also be uniform and point radially outwards; due to cylindrical symmetry, the current will be radial throughout the film. The same total current must be carried out through the film edge and thus

$$\boldsymbol{j}_2 = \frac{I}{2\pi r_2}\hat{\boldsymbol{r}}\,.$$

Assuming a uniform current across the film thickness, we have a radially dependent current distribution,

$$\boldsymbol{j}_2(r) = \frac{I}{2\pi r t}\hat{\boldsymbol{r}}\,,$$

which again is valid up to the vicinity of the junction with the wire. In the film, we can now calculate the Joule heating power per unit volume, P, as a function of r:

$$P_2 = \boldsymbol{j}_2 \cdot \boldsymbol{E}_2(r) = \frac{j_2(r)^2}{\sigma_2} = \frac{I^2}{(2\pi r t)^2 \sigma_2} \,.$$

This decreases as the inverse square of the distance away from the wire and is larger for lower conductivity. The integral power dissipated in the range $r_1 < r < r'$,

$$P_{\text{film}} = (2\pi t) \int_{r_1}^{r'} P_2(r) r \, \mathrm{d}r = \frac{I^2}{2\pi t \sigma_2} \ln\left(\frac{r'}{r_1}\right) \,,$$

By comparison, the Joule heating per unit volume in the wire is uniform:

$$P_1 = \frac{j_1^2}{\sigma_1} = \frac{I^2}{(\pi r_1^2)^2 \sigma_1} \,.$$

Thus, for $r = r_1$,

$$\frac{P_2}{P_1} \approx \frac{1}{4} \frac{\sigma_1}{\sigma_2} \left(\frac{r_1}{t}\right)^2 \,.$$

As a result, the Joule heating in the film (per unit volume) will be significantly greater than that in the wire, in cases where $\sigma_1 \gg \sigma_2$, or where the width of the wire is greater than the thickness of the film. In one of our experimental cases, a gold wire ($r = 12.5\,\mu$m) joins a gold film ($t = 50\,$nm) and the factor difference is $\sim 10^4$, Fig. 3D.

S5. TRACE RESISTOR MODEL

Consider the circuit with a common ground trace shown in fig. S3A. (A trace connecting many elements to a single ground point is a typical, effective method to reduce the total number of electrodes needed to address multi-element arrays.) The array elements are represented by resistances R_1 and R_2, and the ground trace by $R_{\rm X}$ and $R_{\rm Y}$. The circuit is grounded at G, and periodic voltages of equal amplitude are applied to the source electrodes of the elements:

$$V_1 = V_0 \cos(\omega t) \,, \quad V_2 = V_0 \cos(\omega t + \phi) \,, \quad V_{\rm G} = 0 \,,$$

where ϕ is the phase difference between the sources. (The Joule power phase, $\xi = 2\phi$.) Our aim is the find the voltage drop across $R_{\rm X}$, the last resistance in the trace before G. The currents

$$I_1 = \frac{V_1 - V_{\rm X}}{R_1} \,, \quad I_2 = \frac{V_2 - V_{\rm X}}{R_2 + R_{\rm Y}} \,, \quad I_{\rm X} = \frac{V_{\rm X} - V_{\rm G}}{R_{\rm X}} \,.$$

Using Kirchhoff's junction rule, $I_{\rm X} = I_1 + I_2$. The potential across $R_{\rm X}$ is then

$$V_{\rm X} = \frac{V_1 R_{\rm X} R_{2\rm Y} + V_2 R_1 R_{\rm X} + V_{\rm G} R_1 R_{2\rm Y}}{R_{\rm X} R_{2\rm Y} + R_1 R_{\rm X} + R_1 R_{2\rm Y}} \,,$$

where $R_{2\rm Y} \equiv R_2 + R_{\rm Y}$. The power dissipated by each component can then be determined:

$$P_1 = I_1^2 R_1 \,, \quad P_2 = I_2^2 R_2 \,, \quad P_{\rm X} = I_{\rm X}^2 R_{\rm X} \,, \quad P_{\rm Y} = I_2^2 R_{\rm Y} \,.$$

The resulting sound output calculated from equation S1 in the case of a dipole is shown in figs. S3B,C, as a function of trace to element resistance ratio and element separation.

S6. POWER IN THE TRACE

Consider the current $I = I_0 \cos(\omega t)$ through resistance R. The Joule heat dissipated by this resistor,

$$P = I^2 R = \tfrac{1}{2} I_0^2 R \left(1 + \cos(2\omega t)\right) \,.$$

Hence, we get a DC component and sound generation at the second harmonic of the source frequency. The current through resistance $R_{\rm X}$ in the trace resistor model consists of two components:

$$I_{\rm X} = I_{\rm a} \cos\omega t + I_{\rm b} \cos(\omega t + \phi) \,.$$

The power dissipated is

$$\begin{aligned} P_{\rm X} = & \tfrac{1}{2}\left(I_{\rm a}^2 + I_{\rm b}^2\right) R + I_{\rm a} I_{\rm b} R \cos(\phi) \\ & + \tfrac{1}{2} R \left(I_{\rm a}^2 \cos(\omega_2 t) + I_{\rm b}^2 \cos(\omega_2 t + \xi)\right) \\ & + I_{\rm a} I_{\rm b} R \cos(\omega_2 t + \phi) \,. \end{aligned}$$

Therefore, if two elements connected to the trace are driven as an acoustic dipole ($\xi = \pi$, $I_{\rm a} = I_{\rm b}$), $R_{\rm X}$ creates a source with power, $P_{\rm X} = I^2 R(1 + \cos(\omega_2 t + \pi/2))$, which has the same frequency as the two elements but has a phase that differs from both by $\pi/2$.

One way to negate this source is to apply a DC bias, $I_{\rm DC}$, such that

$$I'_{\rm X} = I_{\rm X} + I_{\rm DC} \,.$$

The power dissipated is then

$$P'_{\rm X} = P_{\rm X} + I_{\rm DC}^2 R + 2 I_{\rm DC} R \left(I_{\rm a} \cos(\omega t) + I_{\rm b} \cos(\omega t + \phi)\right) \,.$$

With a DC bias such that $I_{\rm DC} > I_{\rm a,b}$, sound is generated at the first harmonic rather than the second. In this case, by setting $I_{\rm a} = I_{\rm b}$ and $\phi = \pi$, we get

$$P'_{\rm X} = I_{\rm DC}^2 R \,,$$

so the power dissipation in the trace is purely DC, resulting in no sound generation. This elimination of sound in the trace is demonstrated in Fig. 4A.

S7. BRANCH ARRAY

Consider the system shown in fig. S4A. Two branches are terminated by fixed voltages V_1 and V_2 and the third

branch is grounded. From Kirchhoff's junction rule, we know:

$$I_1 + I_2 + I_{\rm g} = 0\,. \tag{S2}$$

The voltages at the terminals and junction point, X, are:

$$V_1 = V_0 \mathrm{e}^{-i\omega t}\,, \quad V_2 = V_0 \mathrm{e}^{-i\omega t}\mathrm{e}^{i\phi}\,,$$
$$V_{\rm g} = 0\,, \quad V_{\rm X} = V_{\rm X0}\mathrm{e}^{-i\omega t}\mathrm{e}^{i\delta}\,.$$

Experimentally, we set V_0 and ϕ. From Ohm's law, the currents are:

$$\begin{aligned} I_1 &= G_1(V_1 - V_{\rm X})\,, \quad I_2 = G_2(V_2 - V_{\rm X})\,, \\ I_{\rm g} &= G_{\rm g}(V_{\rm g} - V_{\rm X}) = -G_{\rm g}V_{\rm X}\,, \end{aligned} \tag{S3}$$

where $G = 1/R$ is the conductance. We can eliminate the unknown $V_{\rm X}$ and its associated phase δ by combining equations S2 and S3:

$$V_{\rm X} = \frac{G_1V_1 + G_2V_2}{\Sigma}\,, \tag{S4}$$

where $\Sigma \equiv G_1 + G_2 + G_{\rm g}$. The currents can now be recast in terms of measurable experimental parameters:

$$\begin{aligned} I_1 &= \left(G_1 - \frac{G_1^2}{\Sigma}\right)V_1 - \frac{G_1G_2}{\Sigma}V_2\,, \\ I_2 &= \left(G_2 - \frac{G_2^2}{\Sigma}\right)V_2 - \frac{G_1G_2}{\Sigma}V_1\,, \\ I_{\rm g} &= -\frac{G_1G_{\rm g}}{\Sigma}V_1 - \frac{G_2G_{\rm g}}{\Sigma}V_2\,. \end{aligned}$$

In the limit $G_{\rm g} \to \infty$:

$$\begin{aligned} &I_1 \to G_1V_1\,, \quad I_2 \to G_2V_2\,, \\ &I_{\rm g} \to -G_1V_1 - G_2V_2\,, \end{aligned}$$

where it is clearly seen that the junction rule holds. Since the currents in each branch involve both V_1 and V_2 we cannot compute the Joule power via IV but only through I^2/G. As such, the powers in the three branches are:

$$\begin{aligned} P_1 =& \frac{I_1^2}{G_1} = \left(1 - \frac{G_1}{\Sigma}\right)^2 G_1V_1^2 + \left(\frac{G_2}{\Sigma}\right)^2 G_1V_2^2 \\ &+ 2\left(\frac{G_1G_2}{\Sigma^2} - \frac{G_2}{\Sigma}\right)G_1V_1V_2\,, \\ P_2 =& \left(\frac{G_1}{\Sigma}\right)^2 G_2V_1^2 + \left(1 - \frac{G_2}{\Sigma}\right)^2 G_2V_2^2 \\ &+ 2\left(\frac{G_1G_2}{\Sigma^2} - \frac{G_1}{\Sigma}\right)G_2V_1V_2\,, \\ P_3 =& \left(\frac{G_1}{\Sigma}\right)^2 G_3V_1^2 + \left(\frac{G_2}{\Sigma}\right)^2 G_3V_2^2 \\ &+ 2\frac{G_1G_2}{\Sigma^2}G_3V_1V_2\,, \end{aligned}$$

which all contain contributions from V_1, V_2 and the combination V_1V_2 (a component of the phantom source). (Note that in the limit $G_{\rm g} \to \infty$, $P_1 \to G_1V_1^2$, as expected.) The total phantom power comprises the V_1V_2 components in each of the branches:

$$P_{12} = -2\frac{G_1G_2}{\Sigma}V_1V_2\,,$$

which is negative (*i.e.* an additional π out of phase with the other heating components). The total Joule power from the 3-branch array:

$$\begin{aligned} P =& P_1 + P_2 + P_3 \\ =& \left(1 - \frac{G_1}{\Sigma}\right)G_1V_1^2 + \left(1 - \frac{G_2}{\Sigma}\right)G_2V_2^2 \\ &- 2\frac{G_1G_2}{\Sigma}V_1V_2\,. \end{aligned} \tag{S5}$$

Generally, if junction X is at the centre of k branches each of conductance G_k then:

$$\begin{aligned} V_X &= \frac{\sum_k G_kV_k}{\sum_k G_k} = \frac{\sum_k G_kV_k}{\Sigma} \\ I_k &= G_k(V_k - V_X) \\ &= \left(G_k - \frac{G_kG_k}{\Sigma}\right)V_k - \sum_{l\neq k}\frac{G_kG_l}{\Sigma}V_l \\ &= (G_k - \Gamma_{kk})V_k - \sum_{l\neq k}\Gamma_{kl}V_l\,, \end{aligned}$$

where $\Gamma_{kl} = \Gamma_{lk} = G_kG_l/\Sigma$. Therefore the power:

$$\begin{aligned} P_k =& G_k^{-1}\left((G_k - \Gamma_{kk})V_k - \sum_{l\neq k}\Gamma_{kl}V_l\right)^2 \\ =& G_k^{-1}\left((G_k - \Gamma_{kk})^2V_k^2 - 2\sum_{l\neq k}(G_k - \Gamma_{kk})\Gamma_{kl}V_kV_l\right. \\ &\left.+ \sum_{l,m\neq k}\Gamma_{kl}\Gamma_{km}V_lV_m\right)\,. \end{aligned}$$

If $k = 3$ and $V_3 = 0$ then we see we can recover the 3-branch equations above.

S8. MINIMISING THE JOULE POWER

From equation S5, if $V_1 = V_1\mathrm{e}^{-i\phi/2}$ and $V_2 = V_2\mathrm{e}^{i\phi/2}$,

$$\begin{aligned} P =& \left(1 - \frac{G_1}{\Sigma}\right)G_1V_1^2\mathrm{e}^{-i\phi} + \left(1 - \frac{G_2}{\Sigma}\right)G_2V_2^2\mathrm{e}^{i\phi} \\ &- 2\frac{G_1G_2}{\Sigma}V_1V_2\,. \end{aligned} \tag{S6}$$

By considering the total absolute Joule power $|P| = \sqrt{PP^*}$, we can minimise this with respect to ϕ by setting

$$\frac{\mathrm{d}\,|P|}{\mathrm{d}\phi} = 0\,,$$

which results in

$$\phi = \arccos\left(\frac{G_1}{2\left(G_1+G_3\right)}\frac{V_1}{V_2} + \frac{G_2}{2\left(G_2+G_3\right)}\frac{V_2}{V_1}\right) .$$

So the total Joule power will have a minimum at a particular phase difference between the sources, which is dictated by the ratios of the source voltages and branch resistances, Fig. 4C. In the case $G_1 = G_2 = G_3$ and $V_1 = V_2$,

$$\phi = \arccos\left(\frac{1}{2}\right) = \frac{\pi}{3} .$$

From equation S6, the total power at $\phi = \pi/3$ is zero.

S9. REAL CIRCUIT ANALYSIS

Consider the circuit shown in fig. S4B. Here we take into account the output resistance, r, of the amplifiers used to drive the currents down the branches. As r can be significant compared to the resistances of the elements in the array, it affects the voltages (magnitudes and phases) that drive the currents.

$$\begin{aligned}
\text{At X :}& I_1 + I_2 = I_3 \\
\text{Loop 1 :}& V_1 = I_1(r+R_1) + I_3R_3 \\
\text{Loop 2 :}& V_2 = I_2(r+R_2) + I_3R_3 \\
\text{Nodes :}& V_X = I_3R_3\,,\; V_A = I_1R_1 + V_X\,, \\
& V_B = I_2R_2 + V_X
\end{aligned}$$

From the loop equations, we can determine I_1 and I_2. From the junction rule at X, we can determine V_X:

$$V_X = \frac{1}{\Sigma'}\left(\frac{V_1}{r+R_1} + \frac{V_2}{r+R_2}\right) ,$$

where $\Sigma' = (r+R_1)^{-1} + (r+R_2)^{-1} + R_3^{-1}$. The voltages at nodes A and B are then:

$$\begin{aligned}
V_A &= \frac{R_1}{r+R_1}V_1 + \frac{r}{r+R_1}V_X\,, \\
V_B &= \frac{R_2}{r+R_2}V_2 + \frac{r}{r+R_2}V_X\,,
\end{aligned}$$

where it can be seen that when $r > 0$ there will be a contribution from both V_1 and V_2 to the voltages measured at A and B. (It is the voltages at A and B that form the sources of the currents down each branch of the array.)

If $R_1 = R_2 \equiv R$ and $V_1 = V_2 \equiv V$ then

$$\begin{aligned}
V_A - V_B &= \frac{R}{r+R}\left(V_1 - V_2\right) \\
&= \frac{R}{r+R}V\left(1 - \mathrm{e}^{i\phi}\right) \\
&= 2\frac{R}{r+R}V\sin(\phi/2)\mathrm{e}^{i\left(\frac{\phi}{2}-\frac{\pi}{2}\right)} .
\end{aligned}$$

Measurements of these voltages and the currents down each branch allow us to calculate the Joule power dissipated. This is shown as the green circles in Fig. 4B.

S10. HETERODYNING

If in the 3-branch array, the two source frequencies are different, f_1 and f_2, then from equation S5 it is clear that the Joule power will have frequency components at $2f_1$, $2f_2$ and the heterodynes $f_1 \pm f_2$. Each branch will carry all components, as shown in fig. S4C. From the trigonometric identity,

$$2\cos(\omega_1 t)\cos(\omega_2 t) = \cos((\omega_1 - \omega_2)t) - \cos((\omega_1 + \omega_2)t) ,$$

it can be seen that the sum and difference heterodynes have a phase difference of π between them (Fig. 4D and fig. S4D).

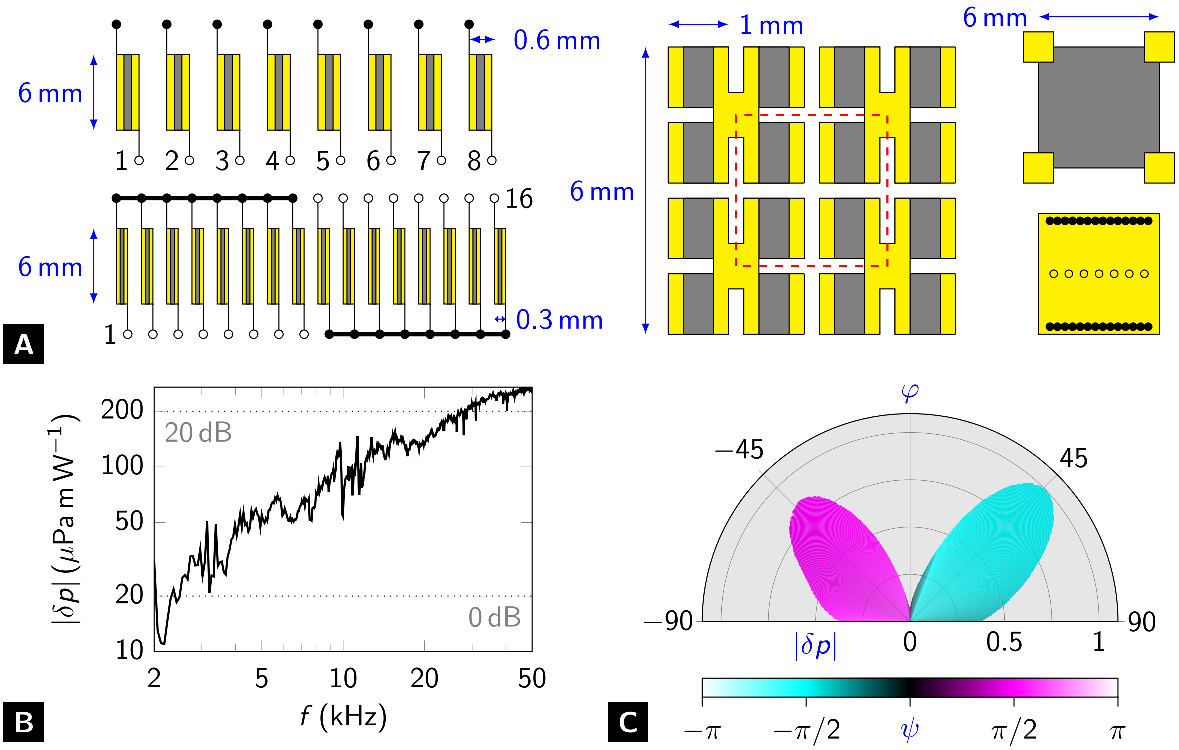

Figure S1. **Thermoacoustic device architecture and basic measurements**. (**A**) Graphene-based 8-element (top left) and 16-element (bottom left) linear arrays (not to scale). For the latter, elements 1-8 share a common ground trace and elements 9-16 share another (thick black lines). For the 8- and 16-element arrays, respectively, the length of the film between gold electrodes (yellow) is 0.2 and 0.1 mm and the pitch between elements is 0.8 and 0.4 mm. (middle) Graphene-based 4×4-planar array, the dashed region indicating the visible region in Fig. 2; (top right) a 4-terminal square ITO film; (bottom right) a 7-element (open circles) gold junction array. The ground for the junction array is a high density of junctions along the top and bottom edge (filled circles). (**B**) A typical sound spectrum from an array element. The sound pressure $|\delta p|$ is shown normalised to 1 m microphone–array separation and 1 W source power. (**C**) Beam profile of the dipolar sound from two array elements measured at 48 kHz and averaged over the range $-90^\circ < \theta < 90^\circ$.

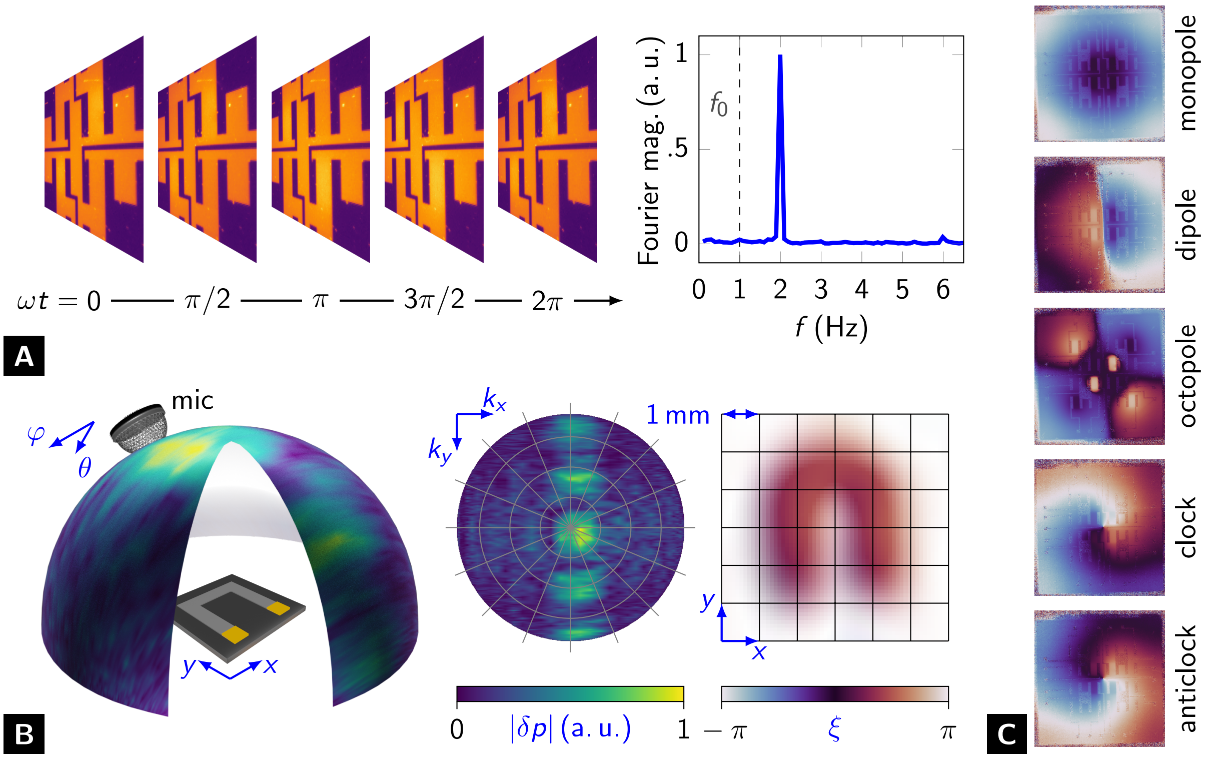

Figure S2. **Fourier transformation of the far-field sound**. (**A**) Time sequence associated with the thermal reconstruction in Fig. 2. The images (left) are shown together with a typical Fourier spectrum (right) of the thermal magnitude at a point on an active element within the array. The electrical source frequency, f_0, is indicated (dashed line). (**B**) Transformation of the sound measured over a hemisphere (left) above a gold-based test device in the form of a horseshoe (side length 3 mm). The orthographic sound projection (middle) is transformed into a (k_x, k_y)-plane. An inverse Fourier transform is performed (right) which returns both the magnitude and phase of the Joule heating on the array surface. (**C**) Further examples of thermal phase reconstruction, as labelled.

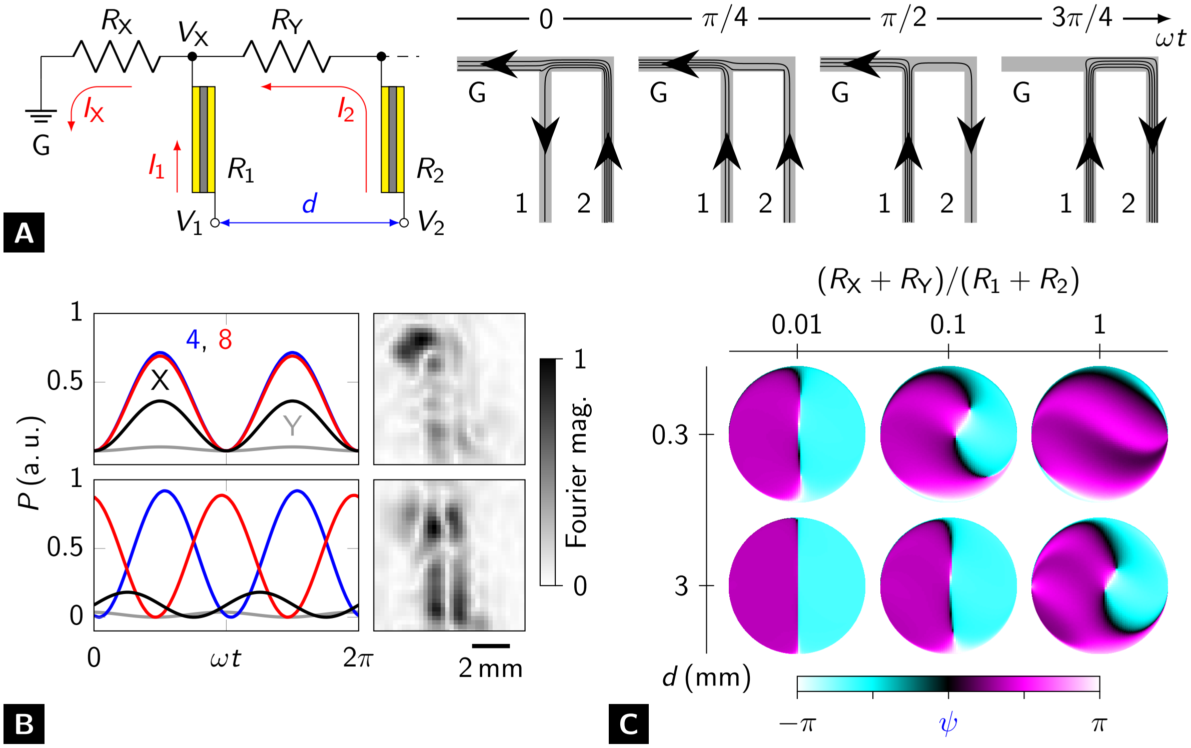

Figure S3. **Features of the common ground trace**. (**A**) Equivalent circuit (left) of two elements (1 and 2) connected to a common ground trace with resistive links (X and Y). The separation, d, between elements is indicated as well as the resistances, R, of each of the resistors in the circuit. (Other annotation refers to the trace resistor model described in supplementary text S5.) Stream plots (right, simulated from numerical model) of the current at different moments in the oscillation of voltage V_1, where V_2 is set with a $\pi/2$ phase lag. Arrows indicate the current direction. (**B**) Elements 4 and 8 of the 16-element array (Supplementary Fig. 1) run together as a monopole (top) and dipole (bottom). The graphs show a model comparison of the power generated in the elements and in the resistive links in the trace ($R_{1,2} = 10$, $R_{X,Y} = 1$). Acoustic reconstructions are shown to the right of each corresponding graph. (**C**) Orthographic projections of the acoustic phase of a simulated 2-element dipolar source as a function of d and relative resistance of the trace to the elements.

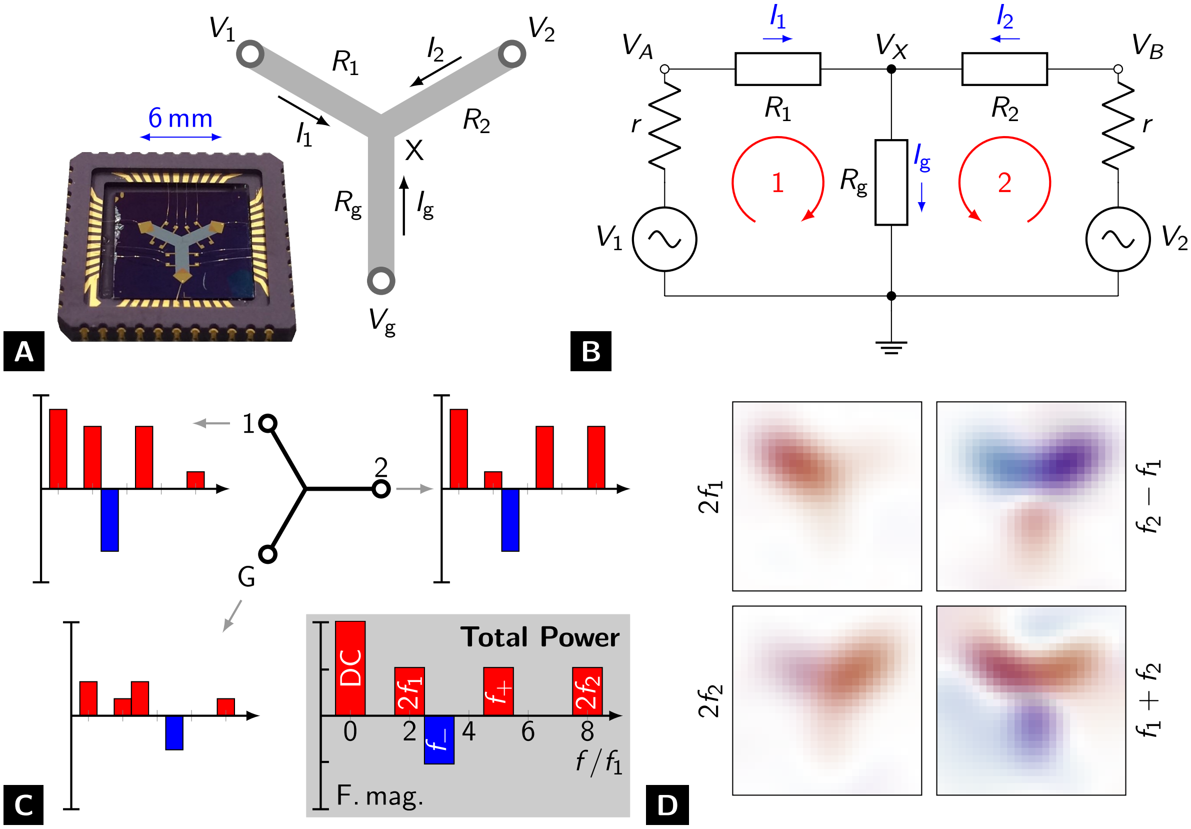

Figure S4. **Features of the phantom source**. (**A**) Photograph of a 3-branch array (left) and a schematic showing parameters used in the modelling (supplementary text S7,8). (**B**) Real circuit model for the 3-branch array, which includes the output resistances, r, of the amplifiers (supplementary text S9). (**C**) Joule power spectral components in each branch of the array for the case where $f_2 = 4f_1$. The source powers occur at $2f_1$ and $2f_2$ and the phantom heterodynes occur at $f_\pm = f_1 \pm f_2$. Negative Joule power corresponds to π radians out of phase. All branch power spectra are shown on the same arbitrary vertical scale. Bottom right: the total Joule power spectrum. (**D**) Experimental acoustic phase reconstructions of sources and heterodynes (cf. Fig. 4D).
